# Psychometric assessment of the Wagnild and Young's resilience scale in Kano, Nigeria

**DOI:** 10.1186/1756-0500-4-509

**Published:** 2011-11-23

**Authors:** Tajudeen Abiola, Owoidoho Udofia

**Affiliations:** 1 Department of Psychiatry, Aminu Kano Teaching Hospital, Kano, Nigeria; 2 Department of Psychiatry, University of Calabar and University of Calabar Teaching Hospital, Calabar, Nigeria

## Abstract

**Background:**

Resilience seemed to lie at the core of the recent promotion of positive mental health and wellbeing. This concept has been well studied in western countries and less in developing countries, particularly Nigeria. The aim of the study is therefore, to demonstrate the internal consistency and concurrent validity of the Resilience Scale (RS) and its 14-item short version (RS-14) in a Nigerian sample.

**Results:**

The RS, RS-14, the Hospital Anxiety Depression Scale (HADS) and two screening questions on experience of recent and upcoming distress were administered to 70 clinical students who consented to participate after a major professional examination. Internal consistency and convergent validity were assessed. The participants mean age was 22.50 years (SD = 0.60). The mean score of RS and RS-14 were 130.23 (SD = 17.08) and 74.17 (SD = 10.14) respectively. Cronbach's alpha coefficient for the RS was 0.87 and that of the RS-14 was 0.81. The mean RS score by gender was 132.04 (SD = 19.08) and 126.52 (SD = 11.50) for males and females respectively and the difference was significant (t = 2.50; p = 0.012). The correlation of RS with RS-14 (r = 0.97; p = 0.000), the HADS depression (r = -0.28; p = 0.017) and anxiety (r = -0.26; p = 0.028) subscales, were significant. The corresponding t-test values for the means of RS and RS-14 scores for both cases and non-cases as determined by HADS, were significant at p < 0.05 and p < 0.01 for the depression and anxiety subscales respectively. The difference between RS means of those who experienced distress (38/125.69) to those that did not (32/134.05) from the recent clinical examination was also significant (t = 2.01; p = 0.045).

**Conclusions:**

The study confirms that the RS and RS-14 may be potentially useful instruments to measure resilience in Nigerians.

## Background

The promotion of positive mental health and wellbeing of populations and communities is an emerging sphere that includes research, policy development, community action and program activity[1]. This according to World Health Organization in a growing global policy focus is to shift the mental health policy and practice from the sole emphasis on services for those suffering from mental illness to the prevention of mental health problems and the promotion of positive mental health[2]. Focusing on concepts such as wellbeing and its promotion redirects health care to recognize the needs of those with and without pathology to develop characteristic traits that delay falling sick and promote quick recovery and remaining so on to flourishing. Developments of indicator scales of psychological wellness are one of the steps towards the actualization of this shift^3^. To this end, several scales have been developed and they measure various aspects of positive mental health for research purpose and in assessing practitioners' impact[3].

The Health Scotland in a review of validated scales grouped positive mental health measures into 8 groups according to the aspects of it they purport to measure[4]. These 8 aspects were emotional wellbeing, life satisfaction, optimism and hope, self-esteem, resilience and coping, spirituality, social functioning, and emotional intelligence^4^. Attainment of all these aspects implies increase quality of life, wellbeing and functional capacity till attainments of resilience to illness and other life adversity[5].

In other words, positive mental health is the attainment of emotional resilience[6]. And resilience is a psychological process developed in response to intense life stressors that facilitates healthy functioning[7]. Furthermore, resilience can be described as the quality that allows an individual or group to function well despite the odds against them[6]. Thus resilience connotes inner strength, competence, optimism, flexibility, and the ability to cope effectively when faced with adversity[5,7].

A core of resiliency and the promotion of mental health is to minimize the impact of risk factors (such as stressful life events) and enhance the protective factors (such as optimism, social support, and active coping) that increase people's ability to deal with life's challenges[6,8,9]. Thus a good measure of resilience may go a long way in the assessment of positive mental health in the context of indicators for mental health planning, policy, research and impact treatment and promotional outcomes. Many measuring scales for the study of resilience have been developed and utilized in western countries. These include the Brief Resilience Scale (BRS)[9], Connor-Davidson Resilience Scale (CDRISC)[10], Baruth Protective Factors Inventory (BPFI)[11], Resilience Scale for Adult (RSA)[12], Brief Resilience Coping Scale (BRCS)[13] and Wagnild and Young Resilience Scale (RS)[14].

A review of some of the above instruments revealed RS as the best instrument to study resilience in adolescents[15]. The RS turned out to be the first instrument developed for the study of resilience as well as one of the most widely used and accurate instruments to measure resilience globally[14,16]. The RS also has been used in many study populations, in several parts of the world, has a 14 item short form (RS-14) and has been translated into other languages [5,14,17-19]. Other benefits include ease of use (6^th ^grade readability), applicability in age group range from adolescent to elderly, and the constructs focus on positive psychological qualities rather than deficits[19].

The above assert that resilience has been well studied in western countries[3-5,15] and less in developing countries, particularly Nigeria. The challenge of moving forward in terms of resiliency in a developing country like Nigeria, and the need to compare our results with other countries should naturally be preceded by the validation of a measuring scale for resilience. Thus, the aim of this study is to demonstrate the internal consistency and concurrent validity of the RS with its short form (RS-14) in a single Nigerian population.

## Methods

### 2.1 Subjects and Procedures

Before the commencement of the study, clearance and permission to conduct the study was obtained from the Ethical and Scientific Committee of Aminu Kano Teaching Hospital, Kano. Participants were 70 out of the 91 students in year 4 clinical medicine of Faculty of Medicine, Bayero University Kano. All the instruments were administered to those who consented to the study, and it took place after a major professional examination.

### 2.2 Instruments of Study

#### i) Wagnild and Young's Resilience Scale

The resilience scale (RS), which measures the capacity to withstand life stressors, and to thrive and make meaning from challenges consists of a 17-item "Personal Competence" subscale and an 8-item "Acceptance of Self and Life" subscale[16]. The short form of RS (i.e. RS-14) is an offshoot of the 25 items and measures similar psychological concept[19]. Resilience as construed by Wagnild comprises of 5 essential characteristics of meaningful life (purpose), perseverance, self-reliance, equanimity and existential aloneness (i.e. coming home to yourself)[19]. The first of these characteristics is identified as the most important that lays the foundation for the other four[19]. The RS and its short version have good validity and reliability from several studies[5,16-19].

#### ii) The Hospital Anxiety Depression Scale

The Hospital anxiety and depression scale (HADS) is a portable easy to administer measure that screens for the presence of anxiety or depressive state in both clinical and non-clinical population. It consists of seven depression items and seven anxiety items and has been validated for use in Nigeria[20]. A score of 8 and above on either of the two components is regarded as case.

#### iii) Two Screening Questions on Experience of Distress

One of the screening questions examines the experience of distress felt from the recently concluded clinical examination and the other on the anticipatory distress that may come with any upcoming major life event. They were simply scored on a 'yes' or 'no' subjective response from participants.

### 2.3 Statistical Analyses

The results were coded and analysed using SPSS 16 statistical package. The Cronbach's alpha coefficient was used to determine the reliability of the RS and RS-14, while the Pearson's correlation coefficient of RS with the RS-14, and the HADS were determined for the concurrent validity. All statistical evaluations were at 2-tailed tests, with p values of less than 0.05 considered as significant.

## Results

### Sociodemographic Variables

Seventy of the 91 clinical students participated in this study. Of the 70 participants, 47 (67.1%) of them were males and majority (64/91.4%) were single. As shown in table 1 the participants mean age was 22.5 years (SD = 0.6).

**Table 1:** Sociodemographic variables of respondents

VARIABLE		N/% (70/100)	
Age group in years	20-24	41 (58.60)	Mean (SD): 22.50 years(0.60)
	25-29	25 (35.70)	Age range: 20 to 34 years
	30-34	4 (5.70)	
			
Gender	Male	47 (67.1)	
	Female	23 (32.9)	
			
Marital status	Single	64 (91.4)	
	Married	6 (8.6)	

### Psychological Variables

The mean and standard deviation of the RS and RS-14 were 130.23 (SD = 17.08) and 74.17 (SD = 10.14) respectively. The corresponding minimum and maximum score range for the RS and RS-14 were 38-160 and 23-90 respectively. The Cronbach's alpha coefficient for the RS was 0.87 and that of the RS-14 was 0.81. The mean RS score by gender was 132.04 (SD = 19.08) and 126.52 (SD = 11.50) for males and females respectively and the difference was statistically significant (t = 2.498; p = 0.012).

The Hospital Anxiety Depression Scale (HADS) screened 22 (31.4%) and 24 (34.3%) of the clinical students as cases for depression and anxiety respectively. The concurrent correlation coefficient of RS with RS-14 (r = 0.970; p = 0.000), HADS depression subscale (r = -0.2.84; p = 0.017), and HADS anxiety subscale (r = -0.263; p = 0.028), were statistically significant (see Table 2). Table 3 showed the mean RS and RS-14 scores for both cases and non-cases and their corresponding t-test values which were significant at p < 0.05 and p < 0.01 for the depression and anxiety subscales respectively.

**Table 2:** Correlation between resilience scale (RS), 14-item resilience scale and other measures

		RS-25	RS-14
VARIABLES	Mean(SD)	r score	r score
HADS depression subscale	5.44 (4.10)	-0.28*	-0.27*
HADS anxiety subscale	5.83 (3.83)	-0.26*	-0.24*
	Note: r = Pearson's correlation and * = p < 0.05		

**Table 3:** HADS caseness and the corresponding RS and RS-14 scores

	N/% (70/100)	MEAN RS SCORE	T-TEST SCORE/P VALUE	MEAN RS-14 SCORE	T-TEST SCORE/P VALUE
HADS DEPRESSION SUBSCALE	CASE (22/31.4)	125.68	T = -2.42*	70.64	T = -2.48*
	NONCASES (48/68.6)	132.31		75.79	
					
HADS ANXIETY SUBSCALE	CASES (24/34.3)	124.17	T = -3.33**	70.50	T = -3.31**
	NONCASES (46/65.7)	133.39		76.09	
		Note * = p < 0.05; ** = p < 0.01			

As shown in table 4 the mean RS value for the 38 who perceived the concluded clinical examination as stressful was 125.69 and that of the 32 who did not see it as stressful was 134.05. The difference between these means was found to be statistically significant (t = 2.007; p = 0.045). However, the mean value for those anticipating upcoming major life stressor to those not, was not statistically significant (t = 0.951; p = 0.342).

**Table 4:** Experience of distress and the corresponding RS scores

	RESPONSE	MEAN RS SCORE	T-TEST SCORE/P VALUE
DISTRESS FROM RECENT STRESSORS	NO (32)	134.05	T = 2.01
	YES (38)	125.69	P = 0.045
			
ANTICIPATING DISTRESS FROM UPCOMING STRESSORS	NO (32)	132.40	T = 0.95
	YES (38)	127.66	P = 0.342

The students who found the examination stressful had significantly higher anxiety and depression scores than their counterpart. The differences between the scores were statistically significant (t = 3.434, p = 0.001, for anxiety; and t = 4.31, p = <0.000, for depression).

## Discussion

The purpose of this study is to determine the reliability and validity of RS and RS-14 in Nigerians as a tool that can assess protective factors or resources[17]. The internal consistency of both the RS and RS-14 is high and its concurrent validity is relatively low. Despite the relatively lower correlation value, this study found resilience as measured by both the RS and RS-14 to be negatively correlated with both depressive and anxiety symptoms. This is in accordance with findings in other studies[5,16-19,21], that demonstrated fair degree of association by their correlation coefficient's values lying within 0.25-0.5. This study thus supported the hypothesis that resilience is inversely correlated with depression, anxiety and by implication other negative emotional states[5,21,22] as well as positive correlation with numerous desired outcomes including physical and emotional wellness[5,23,24].

The mean RS by gender in this study (132.04 versus 126.52 i.e. males versus females) is significant and comparable to the Hunter and Chandler study (132.5 versus 122.5)[25]. And echoing Wagnild[5] concern in her review of RS, it is our belief that the differences between males and females with regard to resilience need to be studied. Also the mean RS value in the current study (130.23) is lower than that of the United States (154.4)[19]. This may be accounted for by the choice of study population who were all students and have just concluded a major professional examination compared to the non-students American population with no such experience. Nonetheless, this study mean value is higher than that of the Japanese mean (111.19)[17] which was also among students (nursing and psychology students).

Resilience was construed by Wagnild[19] as meaningful life (purpose), perseverance, self-reliance, equanimity and existential aloneness (i.e. coming home to yourself). We examined these in our study as quality that can confer protectiveness against developing depressive and anxiety symptoms among our study subjects as shown by the significant finding between HADS determined cases and non-cases. Also, resilience should demonstrate good coping in the presence of stressors which the question on the experience of recent life stressors causing distress tried to explore. The significant difference found between those experiencing distress and those not, supported more the hypothesis of resilience as one promoting inner strength and effective coping when faced with adversity. The other question on upcoming life stressors borders on the concept of anticipating stress which this study showed not to be statistically significant.

The results from this study showed that the Wagnild and Young's RS and its short form (RS-14) can be used to measure the concept of resilience in Nigerians. However, the sampling procedure, the small sample size, the skewed distribution of participants as being highly intelligent, the relatively low concurrent validity and the majority of the participants being unmarried do limit it application. In addition to these limitations was the absence of the utilization of other symptoms or traits scales in this study as well as the non-representativeness of the participants to the multi-ethnic diversity of Nigerians. In other words, this study RS mean score cannot be said to be representative of the various ethnic groups in Nigerians. Despite these deficits, both the RS and its short form will be found handy as constructs that measure resilience on its multidimensionality in further studies. In other words, these measuring tools will serve as a useful tool to help Nigerians explored their individual resilience core in health and illness, know where it is weak and take meaningful steps to strengthen it onto wellness[19,23].

## Conclusions

The researchers do conclude that both the RS and RS-14 have high degrees of internal consistency, relatively low concurrent validity with all the other different measure used in this study, and correlate negatively with psychological distress. All these supported the utility of the RS and RS-14 as potentially useful instruments to assess resilience in the Nigerian populations.

## Competing interests

The authors declare that they have no competing interests.

## Authors' contributions

Both authors conceived the study, design the study, carried out investigation, analyzed data, drafted and critically revised the manuscript.

## References

[B1] Keleher H Armstrong R Evidence-based mental health promotion resource, Report for the Department of Human Services and VicHealth, Melbourne 2005 http://www.health.vic.gov.au/healthpromotion/downloads/mental_health_resource.pdf browsed on 30-10-10

[B2] World Health Organization WHO Mental Health Declaration for Europe; facing the challenges, building the solutions 2005 16250588

[B3] Parkinson J Hogg E Forward; in Parkinson J. Ed. Review of scales of positive mental health validated for use with adults in the UK: Technical report Health Scotland, a WHO Collaborating Centre for Health Promotion and Public Health Development 2007

[B4] Parkinson J Ed Review of scales of positive mental health validated for use with adults in the UK: Technical report Health Scotland, a WHO Collaborating Centre for Health Promotion and Public Health Development 2007

[B5] Wagnild GM a Review of Resilience Scale Journal of Nursing Measurement 2009 17 2 105 13 10.1891/1061-3749.17.2.105 19711709

[B6] (Undated): Glossary of CMHA Mental Health Promotion Tool Kit http://www.cmha.ca/mh_toolkit/intro/pdf/intro.pdf browsed on 30-10-10

[B7] Johnson D Polusny MA Erbes CR King D King L Litz BT Schnurr PP Friedman M Pietrzak RH Southwick SM Resilience and response to stress: Development and initial validation of the Response to Stressful Experiences Scale (RSES) Journal of Affective Disorders 2009 10.7205/milmed-d-10-00258 21366078

[B8] Hamill SK Resilience and Self-Efficacy: The Importance of Efficacy Beliefs and Coping Mechanisms in Resilient Adolescents, Colgate University Journal of the Sciences 2003 http://groups.colgate.edu/cjs/student_papers/2003/Hamill.pdf browsed on 01-09-2011

[B9] Smith BW Dalen J Wiggins K Tooley E Christopher P Bernard J The Brief Resilience Scale: Assessing the Ability to Bounce Back International Journal of Behavioral Medicine 2008 15 194 200 10.1080/10705500802222972 18696313

[B10] Connor KM Davidson JR Development of a new resilience scale: the Connor-Davidson Resilience Scale (CD-RISC) Depress Anxiety 2003 18 2 76 82 10.1002/da.10113 12964174

[B11] Baruth KE Carroll JJ A formal assessment of resilience: The Baruth Protective Factors Inventory The Journal of Individual Psychology 2002 58 235 244

[B12] Friborg O Hjemdal O Rosenvinge JH Martinussen M A new rating scale for adult resilience: What are the central protective resources behind healthy adjustment? International Journal of Methods in Psychiatric Research 2003 12 65 76 10.1002/mpr.143 12830300 PMC6878238

[B13] Sinclair VG Wallston KA The development and psychometric evaluation of the Brief Resilient Coping Scale Assessment 2004 11 94 101 10.1177/1073191103258144 14994958

[B14] Wagnild GM Resilience Scale - A Reliable and Valid tool to Measure Resilience 2009 http://www.resiliencescale.com/browsed on 30-10-10

[B15] Ahern NR Kiehl EM Sole ML Byers J A Review of Instruments Measuring Resilience Issues in Comprehensive Pediatric Nursing 2006 29 103 125 10.1080/01460860600677643 16772239

[B16] Wagnild GM Young HM Development and psychometric evaluation of the Resilience Scale Journal of Nursing Measurement 1993 1 2 165 178 7850498

[B17] Nishi D Uehara R Kondo M Matsuoka Y Reliability and validity of the Japanese version of the Resilience Scale and its short version BMC Res Notes 2010 3 310 Published online 2010 November 17; and accessed on 01-12-10 10.1186/1756-0500-3-310 21083895 PMC2993730

[B18] Girtler N Casari E Brugnolo A Cutolo M Dessi B Guasco S Olmi C De Carli F Italian validation of the Wagnild and Young Resilience Scale: a perspective to rheumatic diseases Clinical and Experimental Rheumatology 2010 20822709

[B19] Wagnild GM the Resilience Scale User's Guide for the US English version of the Resilience Scale and the 14-Item Resilience Scale 2009

[B20] Abiodun OA A validity study of the Hospital Anxiety and Depression Scale in general hospital units and a community sample in Nigeria The British Journal of Psychiatry 1994 165 669 672 accessed on April 9, 2011 10.1192/bjp.165.5.669 7866683

[B21] Humphreys J Research in sheltered battered women Issues in Mental Health Nursing 2003 24 137 152 10.1080/01612840305293 12554425

[B22] Rew L Taylor-Seehafer M Thomas NY Yockey RD Correlates of resilience in homeless adolescents Image: Journal of Nursing Scholarship 2001 33 1 33 40 10.1111/j.1547-5069.2001.00033.x 11253578

[B23] Black C Ford-Gilboe M Adolescent mothers: Resilience, family health work and health-promoting practices Journal of Advanced Nursing 2004 48 4 351 360 10.1111/j.1365-2648.2004.03204.x 15500529

[B24] March M Well being of older Australians: The interplay of life adversity and resilience in late life development Unpublished doctoral dissertation, Charles Sturt University, Australia 2004

[B25] Hunter AJ Chandler GE Adolescent Resilience Image: Journal of Nursing Scholarship 1999 31 2 243 247 10.1111/j.1547-5069.1999.tb00488.x 10528454

